# Primary Tumor Resection Improves Survival in Patients With Metastatic Gastrointestinal Stromal Tumors: A Preliminary Population-Based Analysis

**DOI:** 10.3389/fonc.2020.01440

**Published:** 2020-08-19

**Authors:** Si Zhao, Hanlong Zhu, Ruonan Jiao, Xueru Wu, Xiuhua Zhang, Guozhong Ji

**Affiliations:** Medical Centre for Digestive Diseases, Second Affiliated Hospital, Nanjing Medical University, Nanjing, China

**Keywords:** gastrointestinal stromal tumors, metastasis, primary tumor resection, survival, SEER

## Abstract

**Background:** Surgery has been the primary treatment in patients with localized gastrointestinal stromal tumors (GISTs) for many decades, whereas it remains controversial regarding the efficacy of primary tumor resection for metastatic GISTs treated with chemotherapy, and likewise it is unclear who would benefit from the surgical resection.

**Methods:** GISTs patients with distant metastases were identified from the Surveillance, Epidemiology, and End Results (SEER) database between 2010 and 2016. Cox proportional hazards regression models were used to identify prognostic factors of overall survival (OS) and cancer-specific survival (CSS). Kaplan-Meier analyses and log-rank tests were conducted to assess the effectiveness of surgery on survival.

**Results:** In total, of 455 patients with metastatic GISTs, 235 patients (51.6%) underwent primary tumor resection and 220 patients (48.4%) did not. Median survival of patients in resection group was 72 (95% CI: 62.90–81.10) months vs. 40 (95% CI: 29.53–50.47) months for those in non-resection group (*p* < 0.001). Similarly, surgery in conjunction with chemotherapy led to a favorable impact on survival than chemotherapy alone (OS: 72 vs. 40 months, *p* < 0.001; CSS: 74 vs. 44 months, *p* < 0.001). Multivariable analysis showed that both OS (HR: 0.581, 95% CI: 0.386–0.874, *p* = 0.009) and CSS (HR: 0.663, 95% CI: 0.439–0.912, *p* = 0.042] were dramatically improved in patients with surgical removal of primary site, as well as primary tumor size between 5 and 10 cm, while increasing age was predictive of poorer survival. Stratified analysis revealed that patients with tumor locations in the stomach demonstrated a prolonged survival after surgery, with no significant differential surgical effect between the stomach and small intestine.

**Conclusions:** Our study preliminarily suggests that carefully selected patients with metastatic GISTs might prolong survival after treatment of surgery, especially those with a primary tumor between 5 and 10 cm and a tumor located in the stomach.

## Introduction

Gastrointestinal stromal tumors (GISTs) which derive from the interstitial cells of Cajal or their precursors, are the most frequent mesenchymal neoplasms of the gastrointestinal (GI) tract, with an estimated prevalence of 130 per million and the lowest annual incidence of 4.3 per million (1–3). GISTs usually have morphologically and clinically heterogeneous features, for which they may occur anywhere along the GI tract, ranging from esophagus to rectum, but the most common location is the stomach, approximately accounting for 60–70% (4). It is widely known that up to 80% of GISTs can harbor functional mutations in PDGFRA (platelet-derived growth factor receptor α) or KIT which have the function of encoding receptor tyrosine kinase protein called CD117 antigen. It is these activating mutations that are predominantly responsible for the initiation of the malignant process in GISTs (5). Some studies indicate that the rate of overt metastatic diseases in patients with GISTs is roughly between 15 and 50% with the most common sites for metastases being the liver (6, 7).

Prior to the imatinib era, surgery is the only potentially curative therapy for patients with localized or resectable GISTs, which is also a standard treatment in patients with tumors >2 cm in diameter. Even so, almost three-fifths of patients experience local tumor recurrence and metastasis after radical surgery during follow-up, with small studies suggesting a 5-year survival rate between 28 and 80% (8). Imatinib mesylate (IM), as a tyrosine kinase inhibitor, has been widely demonstrated its efficacy in terms of overall survival (OS) and progression-free survival (PFS), especially for metastatic or advanced GISTs (9, 10). Nevertheless, with the long-term use of IM, most patients will inevitably occur adverse events and secondary resistance to the drug, in part, as a result of secondary molecular alteration (secondary mutations) attributed to high tumor burden (11). Further to this, given the considerable inter-individual variability, some patients may not respond to IM and experience rapid progression, while the beneficial effects of IM can be maintained indefinitely in the other patients (12). For these reasons, as a means of assisted treatment, primary tumor resection is attempted for recurrent and metastatic GISTs on the basis of the theory that cytoreduction can minimize the number of tumor cells exposed to IM, thereby reducing the risk of secondary mutations (13). However, there is no consensus on the efficacy of surgery in patients with metastatic GISTs. Both Mussi et al. and Sato et al. currently do not make any unequivocal recommendations on whether we should perform surgery on the primary site in this clinical scenario since the trials are fraught with multiple limitations (14, 15). As such, additional efforts to ascertain the therapeutic role of surgery for GISTs are needed.

For such a disease process with an insufficient population and relatively low incidence, we use a well-constructed database named SEER (Surveillance, Epidemiology, and End Results) which can potentially offer more detailed and accurate results. The aim of our study was to assess whether and for whom primary tumor resection has a survival benefit on metastatic GISTs patients undergoing chemotherapy, as well as compare the survival outcomes between combined therapy and chemotherapy alone.

## Patients and Methods

### Database and Patient Selection

We conducted a retrospective review of all GIST patients registered in the SEER database from 2010 to 2016. SEER, sponsored by the National Cancer Institute, records information on patient demographics, cancer incidence and prevalence, tumor characteristics, treatments and mortality, which consists of 19 regional registries and comprises ~34% of the population across the USA (16). SEER^*^Stat software (Version 8.3.6; National Cancer Institute, Bethesda, MD, USA) is accessible to us to capture detailed information from the database.

**Figure 1:**
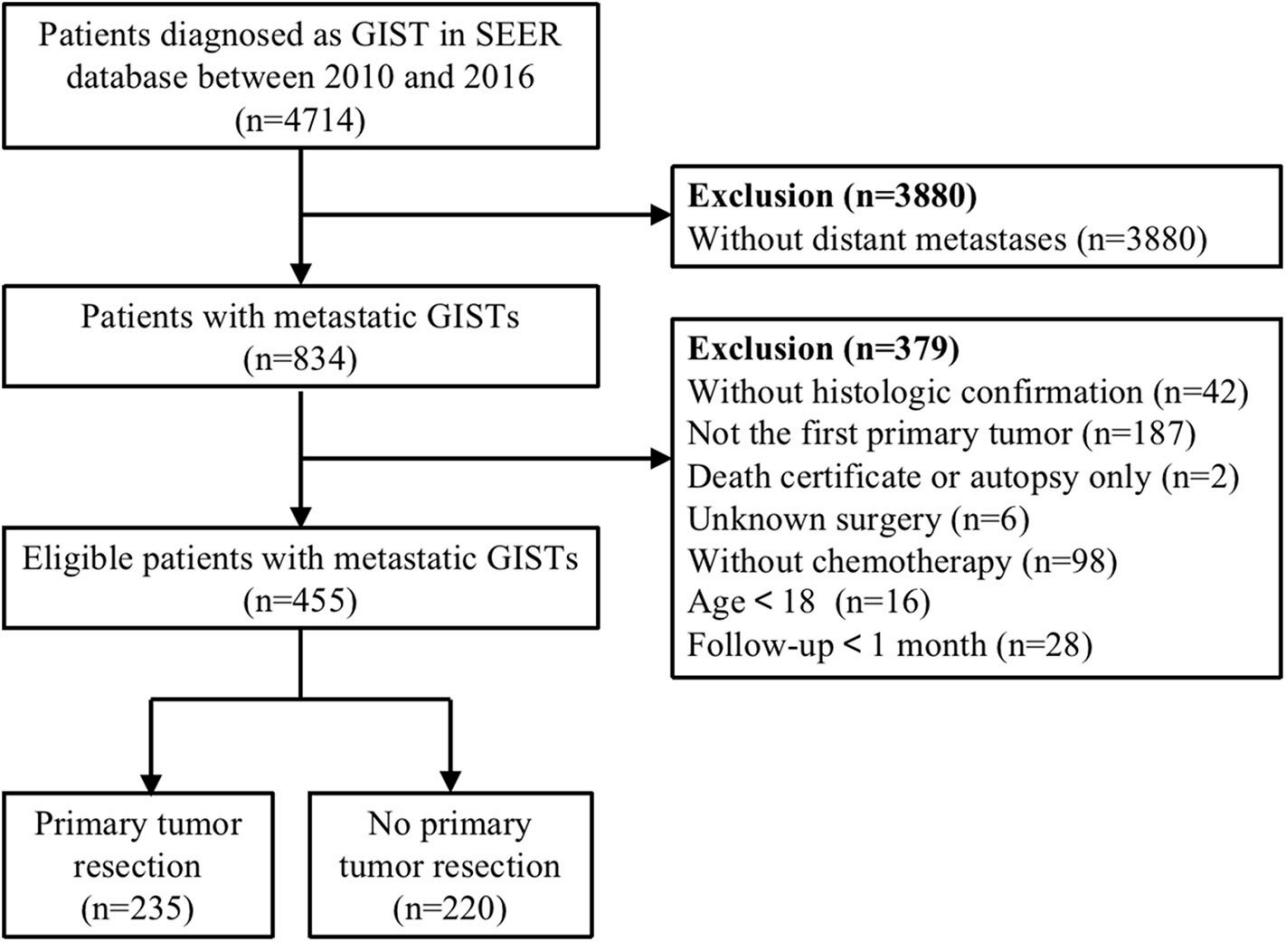
Flow diagram of eligible patients diagnosed with metastatic gastrointestinal stromal tumors (GISTs).

A total of 4,714 cases were initially identified based on the specific ICD-O-3 histologic codes (8936, Gastrointestinal stromal tumor). Then, we excluded patients without distant metastases, chemotherapy, complete data on survival time and surgery, and those who lacked microscopic or histologic confirmation. In addition, patients having a history of another primary malignancy or diagnosed at the time of autopsy were also excluded. After multiple rounds of screening, only 455 patients with metastatic GISTs were enrolled in this dataset. The detailed process of study selection were presented in Figure 1.

### Covariates and Outcomes

Data extraction from the database included the following: demographics (age, race, gender, marital status, and insurance status at diagnosis), cancer characteristics (primary tumor site, tumor size, lymph node status, tumor grade, mitotic index, survival time, vital status, and cause of death), and treatment (primary tumor resection and chemotherapy). Variable “RX Summ—Surg Prim Site” was retrieved to define primary tumor resection, which describes the exhaustive information about surgical removal of the primary lesion by using SEER site-specific codes. Eligible patients were categorized into the resection group and non-resection group. Besides, according to our clinical experience, continuous tumor size variable was grouped into <2, 2–5, 5–10, or>10 cm, with the rest being classified into unknown size. The mitotic index was divided into three groups (≤5/50, >5/50 HPFs or unknown) based on the recognized breakpoint.

OS and CSS were the major endpoint outcomes of our study. We defined OS as the time from a positive diagnosis until death or the last contact date. While CSS was defined as the interval between the date of diagnosis and the date of death attributed to GISTs or the last follow-up. Since the SEER database is public and accessible to applicants, containing unidentifiable patient information, our study was exempted from the approval of the Office of Human Subjects Research of the National Institutes of Health.

### Statistical Analysis

The results were presented as proportions for categorical variables or medians for continuous variables. To identify differences in clinicopathological characteristics among the resection group and the non-resection group, Student's *t*-tests and chi-square tests (or Fisher's exact test) were used for comparisons of continuous variables and categorical variables, respectively. We used the Kaplan-Meier method and the log-rank test to describe the difference in survival between the groups of study patients. Additionally, survival analyses stratified by age, tumor size, primary tumor site and treatment were also performed. Median survival time was reported by using the Kaplan-Meier method. Interaction term analyses (p_int_) in the multivariable model were conducted by age (<65 and ≥65), tumor size (5–10 and >10 cm), and primary tumor site (stomach and small intestine). To gain insight on patient selection for surgery, we conducted a logistic regression model, and the results were expressed as adjusted odds ratios (aORs) and the corresponding 95% confidence interval (CI). For regression survival analysis, we initially established a multivariable Cox regression (named Model 1) which included age, race, gender, marital status, insurance status, tumor site, tumor size, LN metastases, and primary tumor resection. Additionally, to verify the authenticity of our results, a second multivariable model (named Model 2) was carried out based on the least absolute shrinkage and selection operator (LASSO) method proposed by Tibshirani, which was an effective technique for shrinkage and selection method for regression (17). Then, Model 2 was built by incorporating the selected variables with non-zero coefficients from the LASSO regression model (18). Furthermore, Akaike Information Criterion (AIC) and the Bayesian Information Criterion (BIC) were calculated to evaluate the quality of Model 1 in comparison to Model 2, and the best model was defined as the one with the lowest AIC and BIC (19). It is worth noting that we excluded the two variables of mitotic index and tumor grade from the multivariable model because the proportion of unknown data was very high (up to 65%) which would severely reduce the sample size and statistical power for multivariable analysis. In the above analysis, the following software was applied: SPSS 24.0 (IBM Corp, Armonk, NY) for Student's *t*-test, chi-square test, interaction test, and Cox regression analyses, GraphPad Prism 8.3 (GraphPad Software, San Diego, CA) for Kaplan-Meier survival curves and log-rank test, Stata 14.0 (StataCorp, College Station, TX) for forest plot, AIC and BIC, and R 3.6.2 (Institute for Statistics and Mathematics, Vienna, Austria) for LASSO regression. All *P*-values were two-tailed, and *P* < 0.05 was recognized as statistically significant.

## Results

### Patient and Tumor Characteristics

The demographic and clinical features of the participants were summarized in Table 1. In total, 455 patients with metastatic GISTs were considered qualified between 2010 and 2016, of whom 235 (51.6%) underwent primary tumor resection and 220 (48.4%) did not. The mean age at diagnosis was 61 years ranged from 17 to 88 years old. Male patients (60.7%) presented a higher proportion as compared to female (39.3%). The majority of the population were white (71.6%), married (76.5%) and insured (91.4%) individuals. Among these patients, over half were located in the stomach (56.9%), followed by the small intestine (34.1%) and far less frequently in the other sites (5.5%) including esophagus, appendix, peritoneum or retroperitoneum, and colorectum (3.5%). In addition, 14.9% of patients were identified with lymph node metastases. Compared to patients who did not accept surgery, patients in the resection group tended to be younger (59 vs. 62 years, *p* = 0.040), had a greater percentage of tumors ≥5 cm in diameter (88.9 vs. 58.7%, *p* < 0.001), and had more common sites in the small intestine (51.9 vs. 15.0%, *p* < 0.001). Besides, a total of 150 (33%) deaths occurred during follow-up, of which 139 (31%) patients died owing to the metastatic GIST. According to Tables 2, 3 showing the detailed deaths in each group, we can find that the number of deaths was adequate when performing the relevant survival analysis.

**Table 1:** Baseline characteristics of patients with metastatic gastrointestinal stromal tumors.

**Variables**	**Total *n* = 455**	**No primary tumor resection *n* = 220**	**Primary tumor resection *n* = 235**	***p*-value**
Age (years), mean	61.13 ± 14.10	61.51 ± 13.62	59.09 ± 13.72	0.040
Age group, years, %				0.029
<65	288 (63.3)	128 (58.2)	160 (68.1)	
≥65	167 (36.7)	92 (41.8)	75 (31.9)	
Race, %				0.008
White	326 (71.6)	144 (65.5)	182 (77.4)	
Black	72 (15.8)	46 (20.9)	26 (11.1)	
Others	57 (12.5)	30 (13.6)	27 (11.5)	
Gender, %				0.495
Male	276 (60.7)	137 (62.3)	139 (59.1)	
Female	179 (39.3)	83 (37.7)	96 (40.9)	
Marital status, %				0.272
Married	348 (76.5)	166 (75.5)	182 (77.4)	
Unmarried	87 (19.1)	47 (21.4)	40 (17.0)	
Unknown	20 (4.4)	7 (3.2)	13 (5.5)	
Insurance status, %				0.613
Insured	416 (91.4)	199 (90.5)	217 (92.3)	
Uninsured	26 (5.7)	15 (6.8)	11 (4.7)	
Unknown	13 (2.9)	6 (2.7)	7 (3.0)	
Primary tumor site, %				<0.001
Stomach	259 (56.9)	162 (73.6)	97 (41.3)	
Small intestine	155 (34.1)	33 (15.0)	122 (51.9)	
Colorectum	16 (3.5)	10 (4.5)	6 (2.6)	
Other	25 (5.5)	15 (6.8)	10 (4.3)	
Primary tumor size, %				<0.001
≤2 cm	7 (1.5)	5 (2.3)	2 (0.9)	
2–5 cm	39 (8.6)	24 (10.9)	15 (6.4)	
5–10 cm	129 (28.4)	51 (23.2)	78 (33.2)	
≥10 cm	209 (45.9)	78 (35.5)	131 (55.7)	
Unknown	71 (15.6)	62 (28.2)	9 (3.8)	
LN metastases, %				0.278
Yes	68 (14.9)	37 (16.8)	31 (13.2)	
No	387 (85.1)	183 (83.2)	204 (86.8)	
Mitotic index, %				<0.001
≤5/50 HPFs	151 (33.2)	46 (20.9)	105 (44.7)	
>5/50 HPFs	109 (24.0)	15 (6.8)	94 (40.0)	
Unknown	195 (42.9)	159 (72.3)	36 (15.3)	
Grade, %				<0.001
Grade I	25 (5.5)	5 (2.3)	20 (8.5)	
Grade II	42 (9.2)	4 (1.8)	38 (16.2)	
Grade III	30 (6.6)	8 (3.6)	22 (9.4)	
Grade IV	58 (12.7)	10 (4.5)	48 (20.4)	
Unknown	300 (65.9)	193 (87.7)	107 (45.5)	

*Others, including American Indian/AK Native and Asian/Pacific Islander; Other, including esophagus, appendix, and peritoneum/retroperitoneum; LN, lymph nodes; HPFs, high-power fields*.

**Table 2:** Factors associated with overall survival of patients with metastatic gastrointestinal stromal tumors.

**Characteristic**	**Deaths, *n* (%)**	Multivariable (model 1^*^)		**Multivariable (model 2^#^)**	
		**HR (95% CI)**	***p*-value**	**HR (95% CI)**	***p*-value**
Resection of the primary tumor					
No	90 (41)	Reference		Reference	
Yes	60 (26)	0.591 (0.392–0.891)	0.012	0.581 (0.386–0.874)	0.009
Age group					
<65	81 (28)	Reference		Reference	
≥65	69 (41)	1.727 (1.205–2.476)	0.003	1.591 (1.127–2.247)	0.008
Race					
White	104 (32)	Reference			
Black	31 (43)	1.702 (1.099–2.636)	0.017		
Other	15 (26)	0.740 (0.422–1.297)	0.293		
Gender					
Male	85 (31)	Reference		Reference	
Female	65 (36)	1.077 (0.760–1.524)	0.678	1.092 (0.773–1.543)	0.616
Marital status					
Married	121 (35)	Reference		Reference	
Unmarried	26 (30)	0.886 (0.568–1.383)	0.594	0.915 (0.587–1.428)	0.697
Unknown	3 (15)	0.414 (0.120–1.425)	0.162	0.417 (0.131–1.320)	0.137
Insurance status					
Insured	137 (33)	Reference			
Uninsured	11 (42)	1.388 (0.307–6.276)	0.670		
Unknown	2 (15)	2.065 (0.403–10.577)	0.384		
Primary tumor site					
Stomach	87 (34)	Reference		Reference	
Small intestine	40 (26)	0.953 (0.611–1.486)	0.833	0.850 (0.554–1.304)	0.457
Colorectum	5 (31)	1.013 (0.405–2.535)	0.978	0.945 (0.380–2.353)	0.904
Other	18 (72)	1.654 (0.947–2.890)	0.077	1.525 (0.881–2.640)	0.131
Primary tumor size					
≤2 cm	5 (71)	Reference		Reference	
2–5 cm	11 (28)	0.518 (0.168–1.599)	0.252	0.375 (0.125–1.128)	0.081
5–10 cm	32 (25)	0.410 (0.152–1.109)	0.079	0.349 (0.132–0.924)	0.034
≥10 cm	67 (32)	0.600 (0.229–1.575)	0.300	0.497 (0.194–1.276)	0.146
Unknown	35 (49)	0.764 (0.287–2.033)	0.590	0.658 (0.251–1.725)	0.395
LN metastases					
Yes	25 (37)	Reference		Reference	
No	125 (32)	0.934 (0.597–1.460)	0.764	0.992 (0.635–1.547)	0.970

*HR, hazard ratio; CI, confidence interval; LN, lymph nodes*.

* *Model 1: multivariable Cox regression analysis controlling for primary tumor resection, age, race, gender, marital status, insurance status, primary tumor site, primary tumor size, LN metastases*.

# *Model 2: multivariable Cox regression analysis controlling for primary tumor resection, age, gender, marital status, primary tumor site, primary tumor size, LN metastases*.

**Table 3:** Factors associated with cancer-specific survival of patients with metastatic gastrointestinal stromal tumors.

**Characteristic**	**Deaths, *n* (%)**	Multivariable (model 1^*^)		**Multivariable (model 2^#^)**	
		**HR (95% CI)**	***p*-value**	**HR (95% CI)**	***p*-value**
Resection of the primary tumor					
No	81 (37)	Reference		Reference	
Yes	58 (25)	0.667 (0.438–0.946)	0.046	0.663 (0.439–0.912)	0.042
Age group					
<65	75 (26)	Reference		Reference	
≥65	64 (38)	1.733 (1.194–2.515)	0.004	1.686 (1.165–2.439)	0.006
Race					
White	97 (30)	Reference			
Black	29 (40)	1.665 (1.058–2.620)	0.028		
Other	13 (23)	0.700 (0.385–1.273)	0.242		
Gender					
Male	79 (29)	Reference		Reference	
Female	60 (34)	1.068 (0.744–1.533)	0.722	1.077 (0.752–1.542)	0.685
Marital status					
Married	113 (32)	Reference		Reference	
Unmarried	23 (26)	0.842 (0.527–1.348)	0.475	0.875 (0.547–1.400)	0.578
Unknown	3 (15)	0.512 (0.152–1.729)	0.281	0.609 (0.181–2.057)	0.425
Insurance status					
Insured	129 (31)	Reference		Reference	
Uninsured	9 (35)	3.013 (0.382–23.743)	0.295	3.297 (0.412–26.374)	0.261
Unknown	1 (8)	3.782 (0.429–33.367)	0.231	3.920 (0.439–35.034)	0.221
Primary tumor site					
Stomach	82 (32)	Reference		Reference	
Small intestine	36 (24)	0.871 (0.549–1.383)	0.559	0.771 (0.494–1.203)	0.252
Colorectum	4 (25)	0.861 (0.311–2.386)	0.774	0.811 (0.294–2.235)	0.686
Other	17 (68)	1.620 (0.912–2.878)	0.100	1.489 (0.847–2.618)	0.167
Primary tumor size					
≤2 cm	5 (71)	Reference		Reference	
2–5 cm	10 (26)	0.448 (0.141–1.421)	0.173	0.338 (0.108–1.059)	0.063
5–10 cm	30 (23)	0.345 (0.126–0.944)	0.038	0.309 (0.113–0.846)	0.022
≥10 cm	62 (30)	0.502 (0.190–1.328)	0.165	0.442 (0.167–1.171)	0.100
Unknown	32 (45)	0.681 (0.253–1.830)	0.446	0.617 (0.229–1.661)	0.340
LN metastases					
Yes	25 (37)	Reference		Reference	
No	114 (29)	1.058 (0.673–1.662)	0.808	1.104 (0.703–1.734)	0.669

*HR, hazard ratio; CI, confidence interval; LN, lymph nodes*.

* *Model 1: multivariable Cox regression analysis controlling for primary tumor resection, age, race, gender, marital status, insurance status, primary tumor site, primary tumor size, LN metastases*.

# *Model 2: multivariable Cox regression analysis controlling for primary tumor resection, age, gender, marital status, insurance status, primary tumor site, primary tumor size, LN metastases*.

### Influence of Primary Tumor Resection on OS and CSS

**Figure 2:**
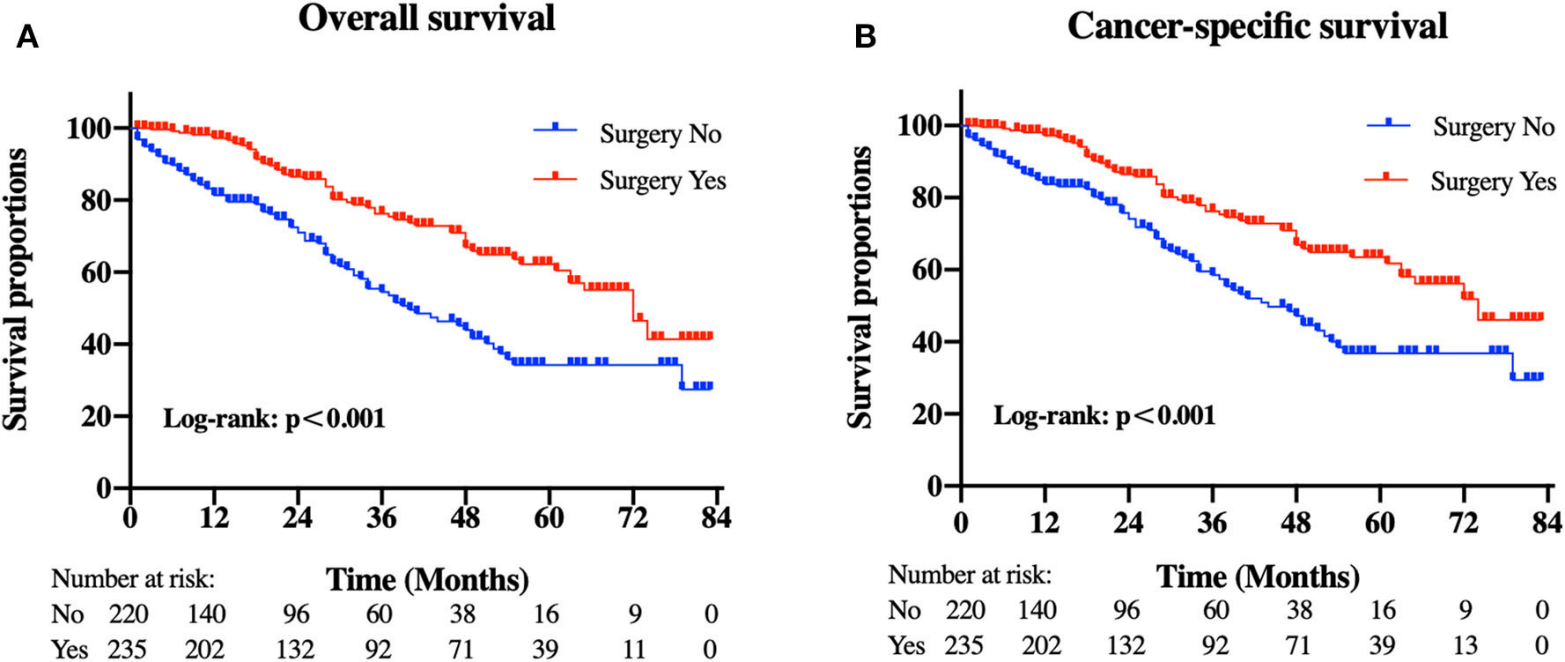
Kaplan-Meier curves of **(A)** overall and **(B)** cancer-specific survival according to whether patients underwent primary tumor surgery in the overall cohort.

**Figure 3:**
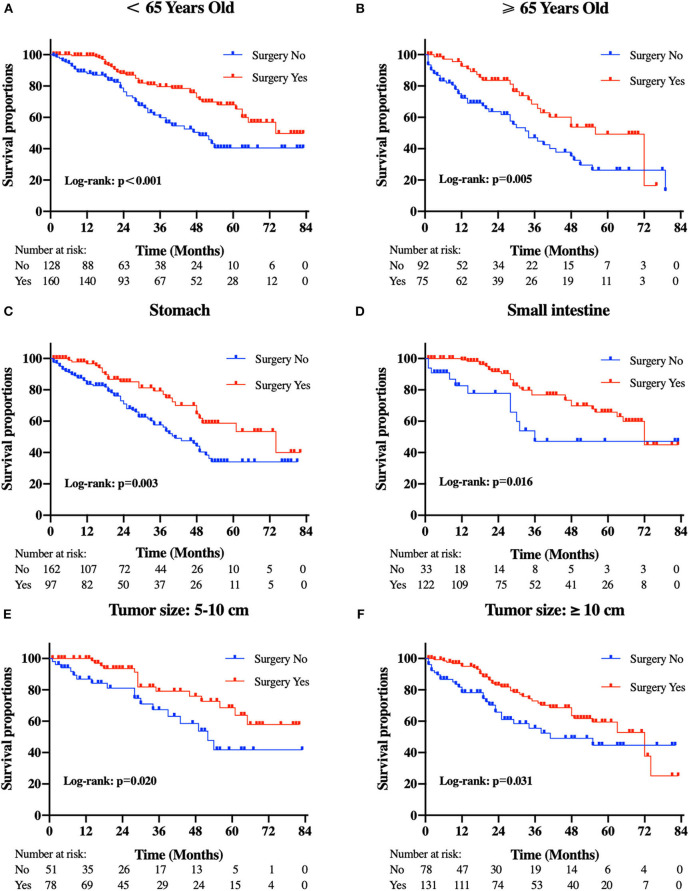
Kaplan-Meier curves of overall survival according to tumor subtype. **(A)** patients <65 years old, **(B)** patients ≥65 years old, **(C)** tumor locations in the stomach, **(D)** tumor locations in the small intestine, **(E)** tumor size between 5 and 10 cm, and **(F)** tumor size ≥10 cm.

**Figure 4:**
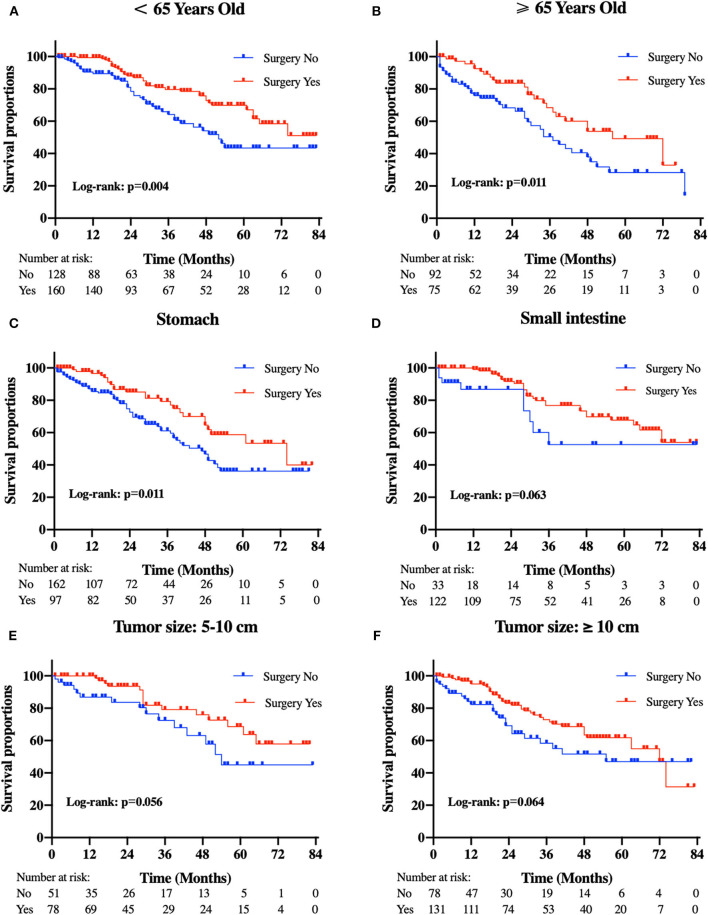
Kaplan-Meier curves of cancer-specific survival according to tumor subtype. **(A)** patients <65 years old, **(B)** patients ≥65 years old, **(C)** tumor locations in the stomach, **(D)** tumor locations in the small intestine, **(E)** tumor size between 5 and 10 cm, and **(F)** tumor size ≥10 cm.

Patients in the resection group were associated with a significantly high likelihood of longer median survival than those without surgery (72 months, 95% CI: 62.90–81.10 vs. 40 months, 95% CI: 29.53-50.47 months, *p* < 0.001). Likewise, there was a trend toward a higher 5-year OS (62.2%, 95% CI: 53.2–71.2% vs. 34.3%, 95% CI: 24.1–44.5%) and CSS (63.5%, 95% CI: 54.4–72.5% vs. 36.8%, 95% CI: 26.2-47.4%) in surgery group compared to patients who did not have primary tumors removed (Figure 2). Moreover, patients were divided into multiple subgroups stratified by age, primary tumor size, and primary tumor site. Our stratification analysis indicated that patients in the surgical cohort were connected with improved OS at 5 years (Age <65 years old: 67.9%, 95% CI: 57.2–78.7% vs. 40.5%, 95% CI: 25.2–55.8%; Age ≥65 years old: 49.3%, 95% CI: 30.1–68.5% vs. 26.3%, 95% CI: 12.6–40.0%; Stomach: 53.4%, 95% CI: 37.1–69.6% vs. 34.1%, 95% CI: 21.2–47.0%; Small intestine: 65.7%, 95% CI: 54.0–77.5% vs. 47.1%, 95% CI: 19.3–75.0%; Tumor size between 5 and 10 cm: 68.6%, 95% CI: 51.8–85.5% vs. 41.8%, 95% CI: 19.8-63.7%; Tumor size >10 cm: 59.4%, 95% CI: 46.9–71.9% vs. 44.7%, 95% CI: 27.8–61.5%) (Figure 3). Similarly, when analyses were conducted separately in the above subgroups, the prolonged 5-year CSS rates in resection group were significantly detected among patients with age older than 65 years old (69.8%), as well as in patients with age younger than 65 years old (49.3%) and patients with tumor arising in the stomach (53.4%), while patients in the corresponding non-resection group had 5-year CSS rates of 43.4, 28.3, and 36.1%, respectively (Figure 4). Of importance, our results showed that surgery in conjunction with chemotherapy led to a favorable impact on survival than chemotherapy alone (OS: 72 vs. 40 months, *p* < 0.001; CSS: 74 vs. 44 months, *p* < 0.001). However, a log-rank test revealed that there were no survival differences between resection and non-resection groups in the following subtypes: tumor originated from colorectum, tumor originated from other sites, tumor size <2 cm, and tumor size between 2 and 5 cm (P>0.05).

**Figure 5:**
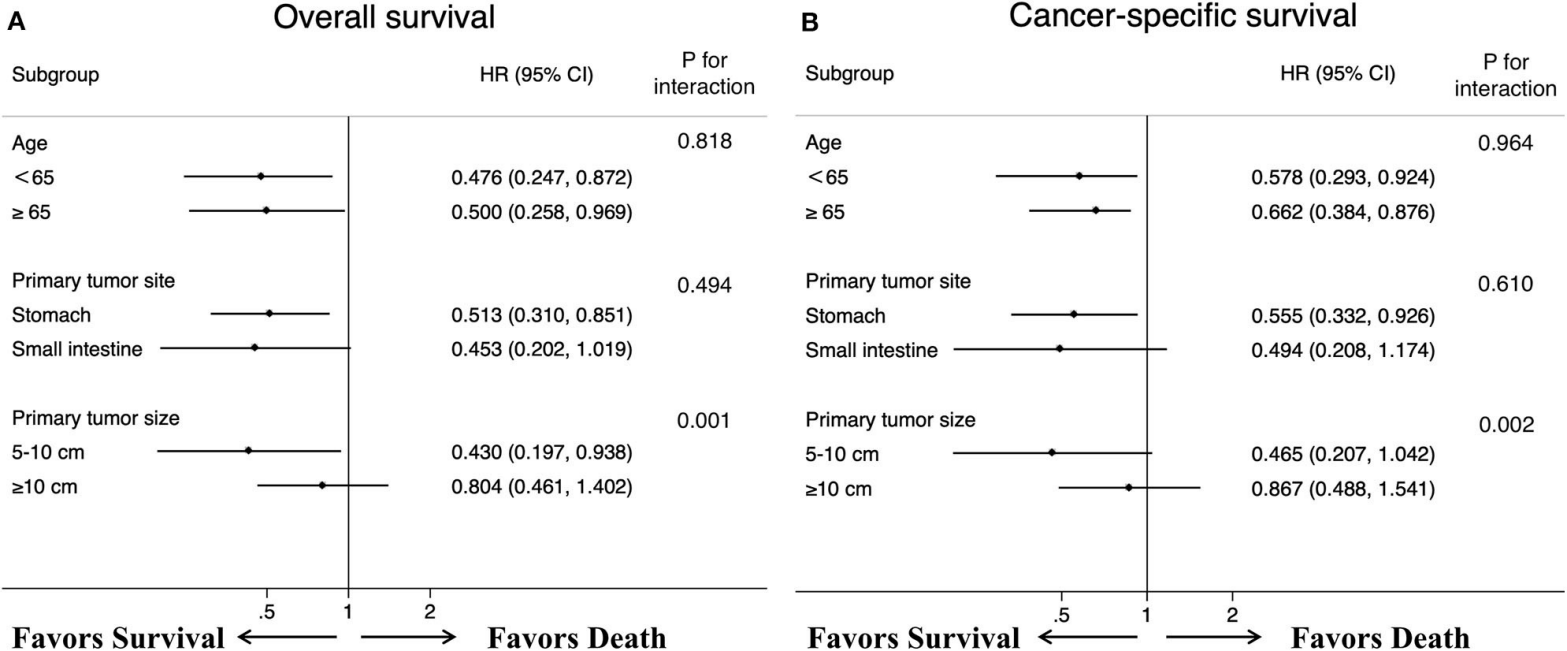
Forest plots summarize the HR and 95% CI of **(A)** overall and **(B)** cancer-specific survival according to whether patients underwent primary tumor surgery in subgroup analyses.

After controlling for confounding factors, removal of primary tumor led to a durable improvement in survival among patients <65 years old (OS HR: 0.476, 95% CI: 0.247–0.872, *p* = 0.032; CSS HR: 0.578, 95% CI: 0.293–0.924, *p* = 0.039) and more than 65 years old (OS HR: 0.500, 95% CI: 0.258–0.969, *p*=0.040; CSS HR: 0.662, 95% CI: 0.384–0.876, *p* = 0.042) (Figure 5). We also found that, a significant correlation between surgery and improved survival was observed in patients with tumor location in stomach (OS HR: 0.513, 95% CI: 0.310–0.851, *p* = 0.010; CSS HR: 0.555, 95% CI: 0.332–0.926, *p*=0.024). A similar improvement was also surprisingly detected across patients with tumor sizes between 5 and 10 cm for OS (HR: 0.430, 95% CI: 0.197–0.938, *p* = 0.034) after treatment with surgery, but not for CSS (HR: 0.465, 95% CI: 0.207–1.042, *p* = 0.063). Regardless of the OS and CSS cohort, there was no difference observed in patients with tumor sizes more than 10 cm or tumor location in small intestine. Specifically, our interaction tests showed that patients who presented with tumor sizes between 5 and 10 cm had a stronger association of surgery with the reduction of overall death or cancer-specific death (p_int_=0.001 for OS; p_int_=0.002 for CSS). The P values for interactions between surgery and age or primary tumor location were not significant (age p_int_ = 0.818 for OS; p_int_ = 0.964 for CSS; primary tumor location p_int_=0.494 for OS; p_int_=0.610 for CSS).

### Multivariable Predictors of Survival

**Figure 6:**
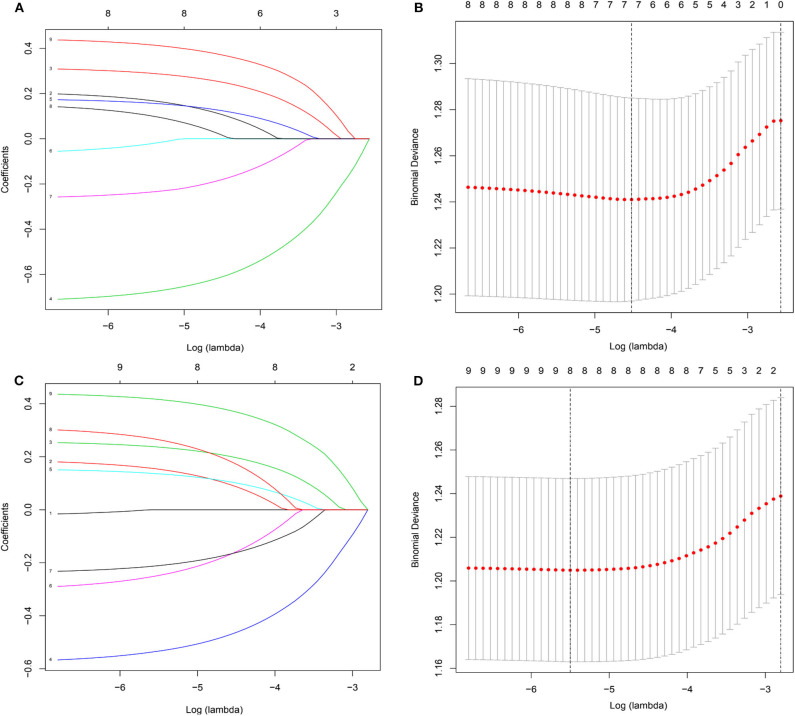
Demographic and clinical feature selection using the LASSO binary logistic regression model. **(A,B)** for overall survival; **(C,D)** for cancer-specific survival.

To gain insight into the association between clinical or tumor characteristics and OS or CSS, a Cox regression was performed. Both in the OS and CSS cohort (Tables 2, 3), multivariable analysis in Model 1 showed that surgery of the primary tumor site was correlated with a decreased risk of overall death (HR: 0.591, 95% CI: 0.392–0.891, *p* = 0.012, Model 1) and cancer-specific death (HR: 0.667, 95% CI: 0.438–0.946, *p* = 0.046, Model 1). On the basis of 455 patients in the cohort, variables were reduced to seven and eight potential predictors for OS and CSS (Figure 6), respectively, which were with nonzero coefficients in the LASSO regression model. Model 2 was adjusted for age, gender, marital status, tumor site, tumor size, LN metastases, and primary tumor resection for OS. Model 2 for CSS was adjusted for age, gender, marital status, insurance status, tumor site, tumor size, LN metastases, and primary tumor resection. After adjustment for confounding factors, the multivariable analysis in Model 2 demonstrated that surgery remained an independent prognostic factor of increased OS (HR: 0.581, 95% CI: 0.386–0.874, *p* = 0.009, Model 2) and CSS (HR: 0.663, 95% CI: 0.439–0.912, *p* = 0.042, Model 2). Moreover, Model 2 had lower AIC (1571.816) and BIC (1600.658) than the Model 1 (AIC: 1575.536; BIC: 1612.619) for OS, indicating better prediction of the model 2, and the same conclusion can be applied to CSS (AIC: 1463.128 and BIC: 1500.211 for Model 1; AIC: 1461.221 and BIC: 1494.183 for Model 2). Therefore, we took Model 2 as the main result. Furthermore, primary tumor size between 5 and 10 cm was also a predictor of decreased hazard of death, whereas increasing age was predictive of poorer survival.

### Logistic Regression on Patient Selection for Surgery

The results of the binary logistic regression comparing no surgery vs. surgery as the outcome of interest were shown in Table 4. Specifically, patients older than 65 years with tumors located in the small intestine were much less likely to perform surgery than other patients, but especially compared to younger counterparts with tumors in the stomach.

**Table 4:** Logistic regression model for receiving surgery.

**Characteristic**	**Adjusted OR (95% CI)**	***p*-value**
Age group		
<65	Reference	
≥65	1.706 (1.060–2.747)	0.028
Race		
White	Reference	
Black	0.716 (0.389–1.316)	0.282
Other	0.952 (0.473–1.915)	0.890
Gender		
Male	Reference	
Female	1.260 (0.799–1.987)	0.319
Marital status		
Married	Reference	
Unmarried	0.746 (0.416–1.338)	0.326
Unknown	1.425 (0.439–4.630)	0.556
Insurance status		
Insured	Reference	
Uninsured	0.708 (0.275–1.821)	0.474
Unknown	1.280 (0.306–5.351)	0.736
Primary tumor site		
Stomach	Reference	
Small intestine	6.340 (3.718–10.811)	0.001
Colorectum	0.883 (0.287–2.718)	0.828
Other	1.060 (0.415–2.707)	0.902
Primary tumor size		
≤2 cm	Reference	
2–5 cm	0.877 (0.122–6.292)	0.896
5–10 cm	3.031 (0.473–19.409)	0.242
≥10 cm	3.604 (0.573–22.652)	0.172
Unknown	0.246 (0.035–1.741)	0.160
LN metastases		
Yes	Reference	
No	0.695 (0.378–1.277)	0.241

*OR, odds ratio; CI, confidence interval; LN, lymph nodes*.

*OR < 1 indicates higher odds of receiving surgery*.

## Discussion

For many decades, GISTs are traditionally regarded as unpredictable and sparse tumors, capable of aggressive behavior. Currently, surgical resection has been established as the sole front-line treatment because of its high insensitivity to conventional standard sarcoma chemotherapy and radiotherapy. For patients with metastatic GISTs, it remains controversial however, if primary tumor removal could confer a survival benefit in the setting of distant metastases (20). With the advances in the cognition of molecular mechanisms, IM has been proposed as principle therapy in the management of metastatic GISTs with a reported overall survival of up to 81 months, representing a paradigm shift in targeted therapy (9, 21). Hence, for the purpose of more intuitively appraising the effectiveness of surgery in the metastatic setting, we conducted the study under the premise that all patients had received chemotherapy.

To our knowledge, this is the largest retrospective study focusing on the survival outcomes of patients who accept surgical resection. Our results provide the initial evidence that primary tumor surgery might yield an association with favorable survival for metastatic GISTs compared with those patients without surgery, and more importantly, this is particularly evident in these patients with a primary tumor between 5 and 10 cm, as well as tumors in the stomach. This information might be beneficial when considering surgical interventions in GISTs patients with distant metastases. Simultaneously, a prospective study taking all potential surgical candidates into account is warranted to confirm our findings.

As for the observed longer survival in patients who had their primary tumors removed, although the exact reasons are not clear, it is mainly because carrying out surgery in primary tumor site for palliative and prognostic aims may contribute to a reduction of tumor load, thereby decreasing secondary gene mutations which confer resistance to IM, ameliorating local tumor-related symptoms and retarding tumor progression (11). From the surgical therapy standpoint, accumulating studies have demonstrated the survival advantage of primary tumor resection in highly selected patients (22, 23). In a retrospective study of 87 patients with advanced GISTs, patients receiving either medical therapy alone or surgical debulking combined with drug therapy had an extension of median OS from 40.4 to 54.8 months (24). Likewise, another single-institution study of 90 advanced GIST patients reached a similar conclusion, emphasizing an increase of overall survival in 38 patients undergoing resection following focal progression with standard doses of IM (53.2 months, CI 36.8–69.6) vs. 52 patients with IM dose escalation alone (35.1 months, CI 25.6–44.7) (25). At the same time, our findings exhibit significantly better survival with median survival time of 72 months in the operation group than previous studies and the discrepancy in outcome seems partially attributed to the different inclusion criteria of patients. It is possible that selection biases may exist in our research because we don't know if these patients receiving resection tend to be relatively good status, which would increase potential confounding effects in the evaluation of the impact of surgery on survival outcomes.

However, consistent with these results, there are other reports, on smaller series, failing to show a benefit for primary tumor resection in the setting of metastatic GIST. We revisit these cohorts to gain further insight into the role of surgical treatment, and this observation could be elucidated by the following. On the one hand, Sato et al. performed their study with small numbers of patients and different frequency of postoperative IM therapy. Only 43% of patients accepting R0/R1 resection and 75% of those who performed R2 resection received this therapy (15). On the other hand, it is worth noting that long-term survival was not observed in advanced patients in whom cytoreductive surgery was carried out prior to IM treatment, which may result in the difference (26). Although few randomized controlled trials (RCTs) concentrating on this point have been conducted, they all ended in failure due to limited patients and poor accrual (27, 28). Hence, from this perspective, there is no doubt that choosing appropriate cases and timing for surgical procedure is the first priority. Analogously, multidisciplinary assessment of these advanced patients using a framework approach should be premeditated before determining different treatment options even if our findings reveal the possible survival benefits of surgery. National Comprehensive Cancer Network (NCCN) guidelines recommend that surgery should be considered for these unresectable or metastatic patients with limited disease progression refractory to IM, advanced tumors responsive to IM, and presented with symptomatic bleeding and obstruction (29). In addition, it is critical to restart chemotherapy as soon as the patient is capable to tolerate oral medication after surgery (30).

On subgroup analysis regarding therapy, as previously shown, patients treated with surgery plus chemotherapy show a tendency of relatively better survival, with 5-year OS rates increasing from 34.3 to 62.2%, when compared with chemotherapy alone, which are supported by recent publications (31, 32). For those who survive to a combination of surgery and chemotherapy, median survival has been demonstrated to be longer in patients with advanced GIST. Notably, contrary to our hypothesis, we found that primary tumor resection seemed to prolong the survival time even among patients with tumor sizes between 5 and 10 cm whereas patients with tumor size smaller than 5 cm did not. This seemingly paradoxical phenomenon might be correlated with the biological behavior of tumors. Generally, patients with smaller tumors are less likelihood of developing metastasis, while once metastases occurring, they would have a higher degree of malignancy and more extensive metastatic diseases over time, which is detrimental to surgery. Besides, the fact that surgical removal supported a negative impact of surgery for patients with tumor sizes >10 cm was detected. Presumably, one possible explanation is that large tumors appear to contain more resistant cells, having a greater chance of recurrence and emerging new resistance mutations during treatment (15). Interaction tests show a differential surgical effect between the two subtypes, further demonstrating a survival benefit of surgery in patients with a tumor size between 5 and 10 cm, which is also perceived as an independent predictive indicator for prolonged OS and CSS in our analyses. Of note, considering the nature of respective analysis, studies in the form of RCTs with balanced characteristics are urgently needed to prove the efficacy of surgical treatment in patients with GISTs and distant metastases.

Despite it is widely recognized that small intestinal GISTs exhibit more aggressive features and portend worse outcomes than gastric GISTs (33), a statistically significant difference regarding OS and CSS was not observed among patients with stomach GIST and patients with small intestine GIST in our study. The growing use of surgery and chemotherapy in patients with small intestine GIST, which could confer a survival improvement, may explain this finding (34). These patients with tumor located in the stomach might have been considered to benefit from their primary tumor resection according to our survival analysis and interaction results. Additionally, younger patients (<65 years old) and older patients (≥65 years old) simultaneously showed a marginal trend toward a favorable survival duration when they perform surgery, and the finding is supported by interaction tests in this subgroup analysis. However, similar to previously published reports (35), our multivariable analysis suggests that older age was an important prognostic factor that could increase the risk of overall death or cancer-specific death. This observation might be the result of poorer physiologic reserves and capability to sustain more invasive treatment in the elderly. Thus, the feasibility of surgery in older patients still needs further verification.

Although the optimal way to reduce or eliminate selection bias is RCTs, such trials always had to be terminated early as a result of lower participant accrual, moral problems, and the rarity of GISTs during the design and implementation of RCTs (36). Accordingly, we did a lot of effort to describe the association and the influence of surgery on survival, including utilizing multiple multivariable Cox regression methods to identify prognostic parameters, performing survival analyses stratified by age, tumor size and tumor site to assess the role of surgery in specific populations, and conducting interaction analysis to explore the stability of our outcomes. Thus, it appeared to be unlikely that such efficacy was completely the result of unadjusted confounding.

There are some limitations that should be acknowledged here. Firstly, given the retrospective design of our study based on the SEER database, it is inevitable to have intrinsic selection bias as clinicians are prone to choose patients for surgery with better performance status, low metastatic extent, and long-lasting good response to therapy, which was also a major limitation. We found that some important factors including age, tumor site, tumor size, mitotic index, and grade were unbalanced between groups, and our binary logistic regression further indicated that younger patients with tumor originated from the stomach were apt to select surgery, thereby weakening the effects of the observed results. Secondly, tumor grade and mitotic index were excluded from our multivariable analysis owing to their large percent of missing data, which were once defined as prognostic factors in previous reports (37). Besides, our study was conducted with a small subset of patients, which might lead to our observations. Hence, larger-scale and well-designed clinical trials are warranted to validate the role of surgery in the multidisciplinary management of GISTs. In addition, some important parameters such as clinical symptoms, KIT gene, exon mutation, detailed chemotherapy information, the timing of surgery, and surgical margin status were not included because they were not available in the SEER registry, and which may have contributed to our findings. Finally, information about local or distant recurrence was not provided; therefore, further analyses are urgently needed before conclusions can be applied to recurrent populations.

We believe that this data does not allow an unequivocal recommendation for surgery in metastatic GISTs patients due to the existence of the above limitations but that it might support a surgical approach in carefully selected patients, especially with tumor sizes between 5 and 10 cm and tumors located in the stomach. Given that it is a preliminary research, our results should be further validated by prospective multicenter collaborative studies with larger samples that examine surgical benefits for patients with metastatic GISTs to obtain high-quality evidence.

## Conclusion

Ultimately, our study preliminarily demonstrates that resection of primary tumors might have a beneficial effect in cases of well-selected patients with metastatic GISTs, especially those with a primary tumor between 5 and 10 cm and a tumor located in the stomach. Moreover, surgical resection combined with chemotherapy could dramatically improve the prognosis of patients compared with chemotherapy alone. We hope that individualized treatments in patients with metastatic GISTs should be carefully designed by multidisciplinary teams. Future prospective trials with large samples, however, are needed to validate these findings because we are unable to determine the burden of metastasis or the decision-making factors for tumor removal in these patients.

## Data Availability Statement

Publicly available datasets were analyzed in this study. This data can be found here: Surveillance, Epidemiology, and End Results (SEER) database (https://seer.cancer.gov/).

## Author Contributions

SZ and HZ collected and analyzed the data and wrote the paper. RJ and XW performed quality assessment and analyzed the data. GJ and XZ conceived and designed this study. All authors reviewed the paper, read, and approved the final manuscript.

## Conflict of Interest

The authors declare that the research was conducted in the absence of any commercial or financial relationships that could be construed as a potential conflict of interest.

## Abbreviations

GISTs: gastrointestinal stromal tumors
SEER: the Surveillance, Epidemiology, and End Results
OS: overall survival
CSS: cancer-specific survival
GI: gastrointestinal
PDGFRA: platelet-derived growth factor receptor α
IM: Imatinib mesylate
PFS: progression-free survival
ICD-O-3: International Classification of Diseases for Oncology, 3rd Edition
aORs: adjusted odds ratios
HR: hazard ratio
CI: confidence interval
LASSO: least absolute shrinkage and selection operator
AIC: Akaike Information Criterion
BIC: Bayesian Information Criterion
RCTs: randomized controlled trials
NCCN: National Comprehensive Cancer Network.
